# Intereleukine-6 and Interleukine-1β levels in post-traumatic stress disorder, depression and healthy controls: a preliminary report

**DOI:** 10.1192/j.eurpsy.2024.1419

**Published:** 2024-08-27

**Authors:** V. Dell’Oste, S. Fantasia, D. Andreoli, V. Pedrinelli, L. Palego, L. Betti, G. Giannaccini, C. Carmassi

**Affiliations:** ^1^Department of Clinical and Experimental Medicine; ^2^Department of Pharmacy, University of Pisa, Pisa, Italy

## Abstract

**Introduction:**

Patients with Post-traumatic stress disorder (PTSD) or mood disorders, as depression, often showed dysregulation of the hypothalamic-pituitary-adrenal axis and autonomic nervous system, resulting in increased levels of pro-inflammatory cytokines and heightened activity of the immune system that may cause alterations in the structure and function of brain regions through direct neurotoxic effects, oxidative stress, changes in levels of neurotransmitters and decreasing some neurotrophins. Among the most studied pro-inflammatory cytokines in this field there are Intereleukine-6 (IL-6) and Interleukine-1β (IL-1β); however, scant and conflicting data are currently available in the literature about their use as potential biomarkers, and even less on possible comparisons in PTSD and depression.

**Objectives:**

The aim of the present study was to evaluate circulating levels of IL-6 and IL-1β in patients with PTSD and to compare them with those of subjects with depression and healthy controls.

**Methods:**

A sample of 45 subjects, including 15 subjects diagnosed with PTSD (PTSD group), 15 with depression (DEP group), and 15 healthy controls (HC group) were recruited at the Psychiatric Clinic of the Department of Clinical and Experimental Medicine, University of Pisa. HC group included subjects recruited on a voluntary basis. The psychiatric diagnosis was assessed by the Structured Clinical Interview for DSM-5-Clinician Version (SCID-5-CV), the Impact of Event Scale-Revised (IES-R) and the Trauma and Loss Spectrum-Self Report lifetime version (TALS-SR). A peripheral venous blood sample was collected to perform the biochemical assays. The analyses of IL-6 and IL-1β were performed with a dedicated enzyme-linked immunosorbent assay (ELISAs) achieved at the Laboratory of Biochemistry of the Department of Pharmacy, University of Pisa.

**Results:**

No statistically significant gender or age differences emerged in the three groups. There were no statistically significant differences in IL-1β levels among the three groups. Conversely, the PTSD group showed higher levels of IL-6 compared to the DEP and to the HC ones, with a statistically significant difference in the post-hoc analysis among the PTSD and DEP groups with respect to the HC one (p<0.05).

**Conclusions:**

Our results suggest the key role of a chronic low-grade inflammatory state in PTSD and in depression, probably related to a dysregulation in HPA axis and cortisol release, with an increase in proinflammatory cytokines including IL-6 that seemed to be more pronounced in PTSD.

**Disclosure of Interest:**

None Declared

